# Supplementary material for: LAESI mass spectrometry imaging as a tool to differentiate the root metabolome of native and range-expanding plant species
Source: Planta. 2018 Aug 23;248(6):1515–23. doi: 10.1007/s00425-018-2989-4 (PMC6244666; doi:10.1007/s00425-018-2989-4)
Supplement: Supplementary file 1 — Contains Supplementary Table 1 with list of significant metabolites and Supplementary Fig. 1 which displays the ablation spots present on the roots that were selected for further analysis (PDF 227 kb) [file 425_2018_2989_MOESM1_ESM.pdf]

SUPPLEMENTARY FILE

LAESI mass spectrometry imaging as a tool to differentiate the root metabolome of native and range-expanding plant species

Purva Kulkarni*****, Rutger A. Wilschut, Koen J.F. Verhoeven, Wim H. van der Putten and Paolina Garbeva

Netherlands Institute of Ecology (NIOO-KNAW), Droevendaalsesteeg 10, 6708 PB Wageningen, The Netherlands

***Corresponding author:** Purva Kulkarni

Tel.: +31 (0)317 473 511

Email: P.Kulkarni@nioo.knaw.nl

SUPPLEMENTARY TABLE

Supplementary Table 1: Significant metabolites and their respective fold change (log_2(FC)) and *p* values (-log_{10}(*p*)) for native and range expanding pairs *C. jacea* (CJ) vs. *C. stoebe* (CS) and *G. molle* (GM) vs. *G. pyrenaicum* (GP).

CJ vs. CS			GM vs. GP		
m/z	**log_2(FC)**	**-log_{10}(*p*)**	***m/z***	**log_2(FC)**	**-log_{10}(*p*)**
892.2366	2.8154	3.4282	887.111	9.2804	8.7636
837.1989	3.0537	3.2022	885.1207	9.039	7.44
245.094	-7.0019	3.1155	980.8683	8.1151	6.4941
270.964	-5.8824	3.0457	486.52534	-13.033	6.2126
213.12	-6.0788	3.037	1096.874	7.8812	6.1562
352.952	-5.8897	3.0347	492.5648	10.482	5.3132
557.2904	1.3107	1.2492	250.8271	1.2766	2.0428
136.0762	1.8652	1.1594	158.2647	4.8462	1.4655
159.0517	1.1667	1.1396	196.58554	-1.1204	1.455
84.96074	1.65	1.0988	252.3956	1.2045	1.4386
536.1757	1.3311	1.0584	1142.875	3.8741	1.4379
99.00528	1.6044	1.0152	64.50864	-1.0269	1.4038
87.0236	1.7072	1.0096	922.8092	3.3317	1.3522
272.955	-2.013	1.0087	1024.339	3.2395	1.3445
59.02047	1.4073	1.0044	637.3655	1.4521	1.2323
			187.6822	3.8414	1.1946
			92.45683	3.2114	1.1408
			172.38286	-2.1105	1.0878
			859.1226	5.4774	1.0524

SUPPLEMENTARY FIGURE

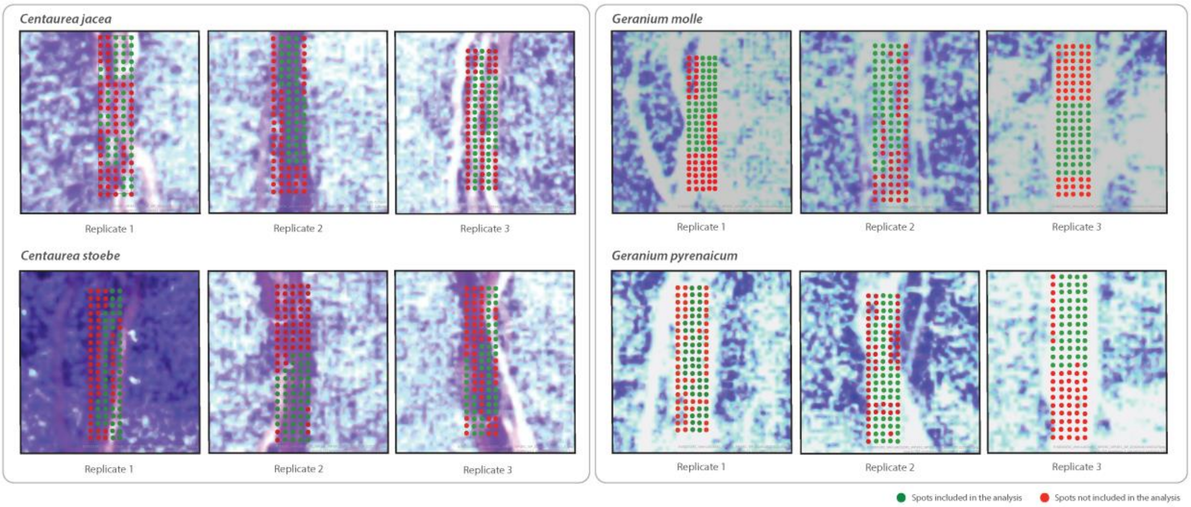

Supplementary Fig. 1 Ablation spots present on the imaged root samples selected for further analysis. A set of 50 ablation spots for each replicate of the native and range expanding species was selected. The spots selected for further analysis are shown in green. These are present on the root sample that has been imaged. The spots that are not selected for further analysis are displayed in red. These may or may not arise from the imaged root samples
